# Optimizing the role of limbal explant size and source in determining the outcomes of limbal transplantation: An *in vitro* study

**DOI:** 10.1371/journal.pone.0185623

**Published:** 2017-09-28

**Authors:** Abhinav Reddy Kethiri, Sayan Basu, Sachin Shukla, Virender Singh Sangwan, Vivek Singh

**Affiliations:** 1 SSR- Stem Cell Biology Laboratory and Center for Ocular Regeneration, L V Prasad Eye Institute, Hyderabad, Telangana, India; 2 Tej Kohli Cornea Institute, L V Prasad Eye Institute, Hyderabad, Telangana, India; 3 Research Scholar, Manipal University, Manipal, Karnataka, India; Cedars-Sinai Medical Center, UNITED STATES

## Abstract

**Purpose:**

Simple limbal epithelial transplantation (SLET) and cultivated limbal epithelial transplantation (CLET) are proven clinical techniques for treating limbal stem cell deficiency (LSCD). However, the ideal size and number of the limbal explants required for transplantation has not been clearly elucidated. This *in vitro* study aimed to determine the optimal limbal explant size required for complete corneal epithelialization by characterizing the cell expansion.

**Methods:**

Limbal explants obtained from both live and cadaveric biopsies were cultured on the denuded amniotic membrane. Explant size and the explant cell outgrowth (expansion) were measured using ImageJ software with respect to days. Cultures were characterized by assessing the rate of proliferation of cells with 5-bromo-2’-deoxyuridine (BrdU) assay along with the expression of different stem cell markers (ABCG2, p63α), corneal epithelial (CK3+12) and adherens junction molecules (E-Cadherin) by immunofluorescence.

**Results:**

Explants from live biopsies had 80% growth potential *in vitro* whereas 40% of the cadaveric tissue failed to grow. Minimum explant sizes of 0.3 mm^2^ for live and ≥0.5 mm^2^ for cadaveric tissue had a mean expansion areas of 182.39±17.06 mm^2^ and 217.59±16.91 mm^2^ respectively suggesting adequate growth potential of the explants. Mean total percentage of proliferative cells was 31.80±3.81 in live and 33.49±4.25 in cadaveric tissue expansion. The expression was noted to be similar in cells cultured from cadaveric compared to cells cultured from live limbal tissue with respect to ABCG2, p63α, CK(3+12) and E-cadherin.

**Conclusion:**

Our findings show that a minimal amount of 0.3 mm^2^ live tissue would be sufficient for ample limbal cell expansion *in vitro*. Cadaveric explants <0.5 mm^2^ had poor growth potential. However, larger explants (≥ 0.5 mm^2^) had growth rate and proliferative potential similar to the live tissue. These findings could prove to be critical for clinical success especially while attempting cadaveric limbal transplantation. This study provides a novel clinical strategy for enhancing efficacy of the limbal transplantation surgery and opens the probability of even using the cadaveric tissue by considering the size of explant.

## Introduction

Epithelium of the cornea undergoes continuous regeneration and renewal through cell proliferation and migration [1, 2]. The source of these cells is usually from the periphery of the cornea [3] where a specialized region known as ‘limbus’ harbors stem cells [4, 5] and their niche [6]. Several conditions such as Aniridia, Steven Johnson’s syndrome, contact lens wear, thermal or alkali injuries may result in vascularization and conjunctivalization of the cornea leading to limbal stem cell deficiency (LSCD) [7]. Limbal transplantation has historically been successful in most of the LSCD cases [8, 9] but, it had also encountered failures in cases of patients who underwent penetrating keratoplasty previously or suffering from persistent dry eye disease [10]. Cell based therapies such as cultivated limbal epithelial transplantation (CLET) [11, 12] and simple limbal epithelial transplantation (SLET)) [13, 14] utilizes limbal biopsy from the healthy eye of the donor from which the progenitor cells originate, facilitating wound healing. Reconstruction of the healthy limbal niche requires not only a good source of limbal cells but also the sufficient size of an explant that supports efficient growth in optimal time after the surgery for better successful outcomes.

The fate of the limbal tissue after transplantation such as the capability of the tissue for cell out growth, its rate of cell expansion, immune insult from the host eye however remains unexplored. Studies on limbal growth properties have been previously reported [15, 16] but, the quantity of limbal tissue to be excised from a healthy eye is not well defined and is presumed to be safe due to the lack of any clinical complications in the donor [9, 17]. For the aforementioned reasons, we speculate that understanding the limbal cell growth and survival of progenitor cells *in vitro* can promote perception and improvement in the present methods of limbal transplantation.

Based on these observations, our objective was addressed by studying the growth properties of the culture in three aspects, which in turn can enhance efficacy of the limbal transplantation technique. Firstly, we explored the expansion capability by measuring the cell outgrowth of the limbal explant cultures that are then statistically compared to that of the average anterior surface area of the human cornea i.e., 132 mm^2^ [18, 19]. Secondly, we enumerated the proliferation rate of the limbal cultures at early and late stages anticipating their ability to proliferate even after transplantation and finally we looked in to the expression of epithelial as well as stem cell markers that represent the heterogeneous pool of corneal and limbal cells in the culture. This is the first study which addressed the role of explant size and the number obtained from different sources (limbal biopsy from living and cadaveric donors) in the growth of a limbal explant in a well characterized *in vitro* model to mimic the limbal transplantation in the patient.

## Materials and methods

### Limbal tissues and study protocol

The study protocol was approved by Institutional Review Board, L. V. Prasad Eye Institute, Hyderabad, India (LEC 04-14-049) and the methodology adhered to the tenets of the Declaration of Helsinki. A total of 20 (n = 20) tissues were evaluated in this study of which 10 (n = 10) live limbal tissues were obtained with written informed consent from the patients undergoing routine CLET/SLET/cataract surgeries (November 2014 to January 2016) and the other 10 (n = 10) tissues were obtained from rejected eyes of the cadaveric donors from Ramayamma International Eye Bank, L V Prasad Eye Institute stored in McCarney Kauffman medium (August 2014 to October 2015). The mean age of the donors was 54.9±10.79 (Range: 35–70) years in live biopsy cases and 45.3±24.55 (Range: 17–85) years in cadaveric cases (S1A Fig). Selection criteria of the cultures *in vitro* were defined as successful if the explant had cell out growth and failure in the case of repeated explant detachment or no cell out growth from the explant even after adherence and five days of culture.

### Processing of human amniotic membrane for scaffold

Human amniotic membranes were processed according to the method previously described by Fatima A *et al*. [20] with a few modifications. Briefly, the membrane was gently peeled off from the nitrocellulose sheet and was placed in a petridish with the epithelium facing up. Further, the membrane was processed by washing with 1x PBS (DPBS powder, D5652-10X1L, Sigma-Aldrich, MO, USA) to remove excess glycerol and then incubated with 0.25% trypsin (T4799-10G, Sigma-Aldrich, MO, USA) and 1mM EDTA (E6758-100G, Sigma-Aldrich, MO, USA) solution at 37°C for 30 minutes for the epithelium to detach. The membrane was then denuded by scraping the epithelium using a cell scraper (3010, Corning, NY, USA). Spent trypsin was discarded and the membrane was washed with 1x PBS after which it was mounted and tucked on a glass slide of appropriate size to obtain a uniform surface. The entire process was performed under sterile conditions.

### Obtaining limbal tissue

For limbal biopsy from live donors, approximately 2 mm^2^ biopsy of superficial limbus was obtained from the donors according to the protocol described by Basu S *et al*. [9] Briefly, a conjunctival flap of 3 mm behind the limbus to be excised was dissected and further proceeding towards the superficial limbus until the excision reached 1 mm in to clear cornea. The biopsy was then collected in the growth medium containing 2x antibiotics and immediately processed for culture.

Limbus from cadaveric tissues was noted for any infections, age of the donor, death to preservation time of the tissue and duration of preservation in eye bank conditions. Tissues were acquired before the date of expiry and incubated for 45 min at 37°C with 2x antibiotics before use. Briefly, the full thickness limbus was excised separating the cornea and scleral tissues with the assistance of Stereomicroscope (SZX10, Olympus, Tokyo, Japan) fitted with an illumination lamp base (SZX2-ILLK, Olympus, Tokyo, Japan). The full thickness limbal rim was further processed by chopping off the lower stromal region to attain superficial limbal explants mimicking the live biopsy tissue acquiring conditions.

### Culturing of human limbal explants

Limbal tissues were chopped in to smaller pieces once obtained from the donor. Each explant was picked up using two 26 gauge needles and gently placed on the denuded amniotic membrane such that one limbal explant was present per amniotic membrane. Orientation of the limbal explants (epithelial/stromal directions) to be placed on the membrane was not taken in to consideration. Later, explants were allowed to sit for half an hour without any medium to facilitate attachment to the amniotic membrane after which the human corneal epithelium (HCE) medium was added to the dish and incubated at 37°C and 5% CO_2_ overnight. The following day, the dish was flooded with HCE media [21] comprising of DMEM/F12 (D0547-10X1L, Sigma-Aldrich, MO, USA) along with 10% Fetal Bovine Serum (FBS) (Gibco, CA, USA), 10 ng/ml human recombinant Epidermal Growth Factor (E9644, Sigma-Aldrich, MO, USA), 5 μg/ml human recombinant Insulin (I2643, Sigma-Aldrich, MO, USA), 100 U/ml Penicillin (P3032-10MU, Sigma-Aldrich, MO, USA) and 100 μg/ml Streptomycin (S9137-25G, Sigma-Aldrich, MO, USA) which can be stored at 4°C for a week. Fresh medium was replaced in the petridish every two days and the culture was maintained until it had become confluent covering up the area of membrane.

### Growth kinetics: Measuring the area of the limbal cell expansion

**Fig 1:**
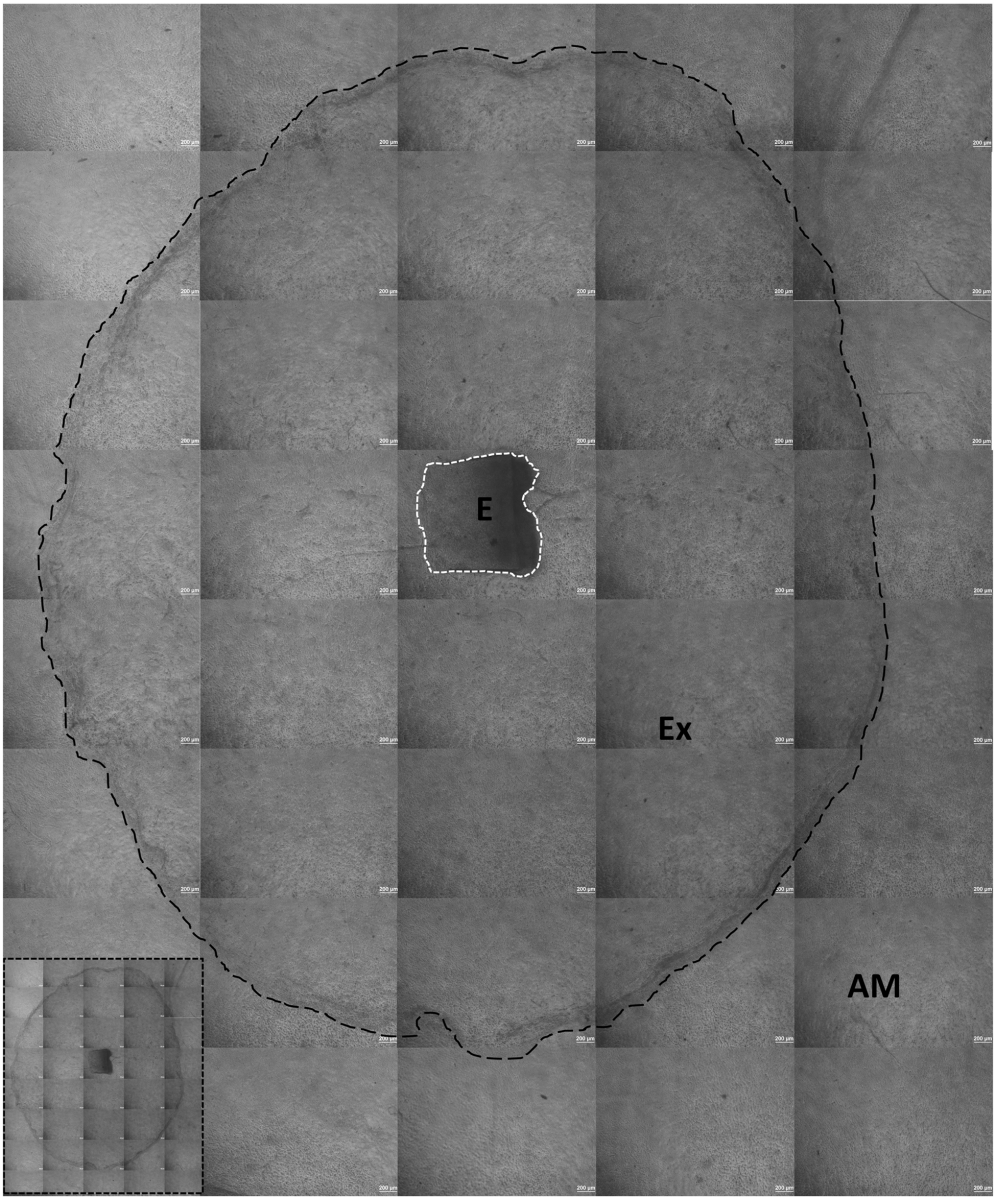
Measuring the area of expansion using Image J. Scale is set initially using the micron bar on the collaged picture. Here, 1μm = 0.415 pixels. White dotted line indicates the area of the explant and black dotted line indicates the outgrowth of cells from explant. AM—Amniotic membrane as scaffold, Ex—Expansion of cells, E—Explant of the limbus. Cell expansion from limbal explant without dotted lines is shown in the inset image. Scale—40X.

The area of the limbal expansion was analyzed by observing the outgrowth of epithelial cells from the limbal explants and recording of images from a phase contrast microscope. The entire two dimensional area of the explant expansion was recorded by capturing several images under 40X magnification of which a collage was prepared in Adobe Photoshop (Version 7.0, Adobe Systems Incorporated, CA, USA). The area of expansion was analyzed by ImageJ (Version 1.50b, National Institute of Health, MD, USA) where the pixel to area conversion was set using the scale bar (Fig 1). The experiment was performed in triplicate for each sample obtained and the subsets were analyzed for the area of expansion.

### BrdU cell proliferation assay

Actively proliferating cells were identified as described by Mariappan *et al*. [22]. Cells were labeled with 5-bromo-2’-deoxyuridine (BrdU) (B5002-100MG, Sigma-Aldrich, MO, USA) which is a nucleotide analog of thymidine. The culture was pulse labeled for 30 minutes by administering BrdU (100 μM/ml) in to HCE growth medium and incubating at 37°C and 5% CO_2_. The culture was then washed thoroughly with 1x PBS before fixing it with 4% Paraformaldehyde (PFA). To detect BrdU incorporated cells in the culture, they were treated with 0.25% Triton-X for 30 minutes followed by 1x PBS wash prior to DNA denaturation with 2N Hydrochloric acid (HCl) (7647-01-0, Thermo Fisher Scientific, Mumbai, India) for an additional 30 minutes. Further, the action of HCl was neutralized by addition of 1 mg/ml sodium borohydride (480886-25G, Sigma, MO, USA) prepared freshly which was washed thrice with 1x PBS before proceeding to immunostaining by anti-BrdU antibody (S1 Table). To assess the proliferation of cells from early (day 3) to late stages (day 9) in the explant culture, we considered 3, 5, 7 and 9 days per sample as time points for BrdU pulse labeling. Photographs of the BrdU stained cells in the culture were obtained near to the explant and along with the periphery of expansion with three random locations at each time point. The mean age of the donors was 55.33±5.03 years (n = 3) and 21.66±4.50 years (n = 3) for live and cadaveric cases respectively.

### Immunofluorescence

Immunofluorescence was performed as reported by Singh V *et al*. [23]. Cells grown on amniotic membrane were fixed with 4% paraformaldehyde for 10 minutes and washed twice with 1x PBS before permeabilization with 0.25% Triton-X (T8787-100ML, Sigma-Aldrich, MO, USA) for 30 minutes. Later, the cells were blocked with 2.5% Bovine Serum Albumin (BSA) (A7096-50G, Sigma-Aldrich, MO, USA) for an hour at room temperature and incubated overnight at 4°C with primary antibody (S1 Table) diluted in 1% BSA. This was followed by 1x PBS wash thrice for 10 minutes and incubation with secondary fluorescence antibody diluted in 1% BSA for 45 minutes which was further washed thrice and mounted on to a glass slide with Fluoroshield Mounting Medium with DAPI (ab104139, Abcam, San Francisco, CA) for nuclei counterstain. Staining of negative controls was done by omitting the primary antibody. Images were documented using an inverted fluorescence microscope (Olympus IX71, Tokyo, Japan) fitted with a camera (Olympus, DP71, Tokyo, Japan) and processed in DP manager software (Version 3.3.1.222).

### Reverse transcription polymerase chain reaction (RT-PCR) analysis

Total RNA was isolated from the confluent limbal cultures (8 days old) using TRIzol reagent (15596018, Invitrogen, Carlsbad, CA) in accordance with the manufacturer’s protocol. RNA was quantified by measuring the absorbance using a spectrophotometer along with the purity evaluation by the ratio of A260/280 (NanoVue^™^ Plus, 28956058, GE Healthcare Bio-Sciences AB, Uppsala, SE). The RNA was further converted to cDNA using reverse transcriptase enzyme (SuperScript III First-Strand Synthesis System, 18080051, Life Technologies, Carlsbad, CA) after which it was subjected to PCR analysis using the primers for ABCG2 (Forward 5’-GGGTTCTCTTCTTCCTGACGACC-3’and reverse 5’-TGGTTGTGAGATTGACCAACAGACC-3’), IL-6 (Forward 5’-ATGAACTCCTTCTCCACAAGCGC-3’and reverse 5’-GAAGAGCCCTCAGGCTGGACTG-3’) and β-Actin (Forward 5’-TCTACAATGAGCTGCGTGTG-3’and reverse 5’-GGTGAGGATCTTCATGAGGT-3’). Initial denaturation was at 95°C for 5 minutes with 35 cycles of denaturation at 95°C for 45 seconds, primer annealing at 55°C for 30 seconds and extension at 72°C for 30 seconds followed by the final extension at 72°C for 5 minutes. PCR products were assessed by 1.5% agarose gel (SeaKem LE Agarose, 50004, Lonza, Basel, CH) under UV illumination (Molecular Imager Gel Doc^™^ XR+ System with Image Lab^™^ Software, 170–8195, Bio-Rad, Hercules, CA). β-Actin was used as an internal control and a 100 base pair ladder (SM0241, Thermo Fisher Scientific, Waltham, MA) was used to evaluate the PCR products.

### Statistical analysis

Statistical analyses were performed using R software (Version 2.12). Data were represented as mean ± standard error (SE) with a significance level set to ‘p’ value of <0.05 with 95% confidence intervals. Comparison among the groups was determined by two sample *t*-tests or linear mixed effect model fit by maximum likelihood wherever required.

## Results

### Live limbal explant cultures

**Fig 2:**
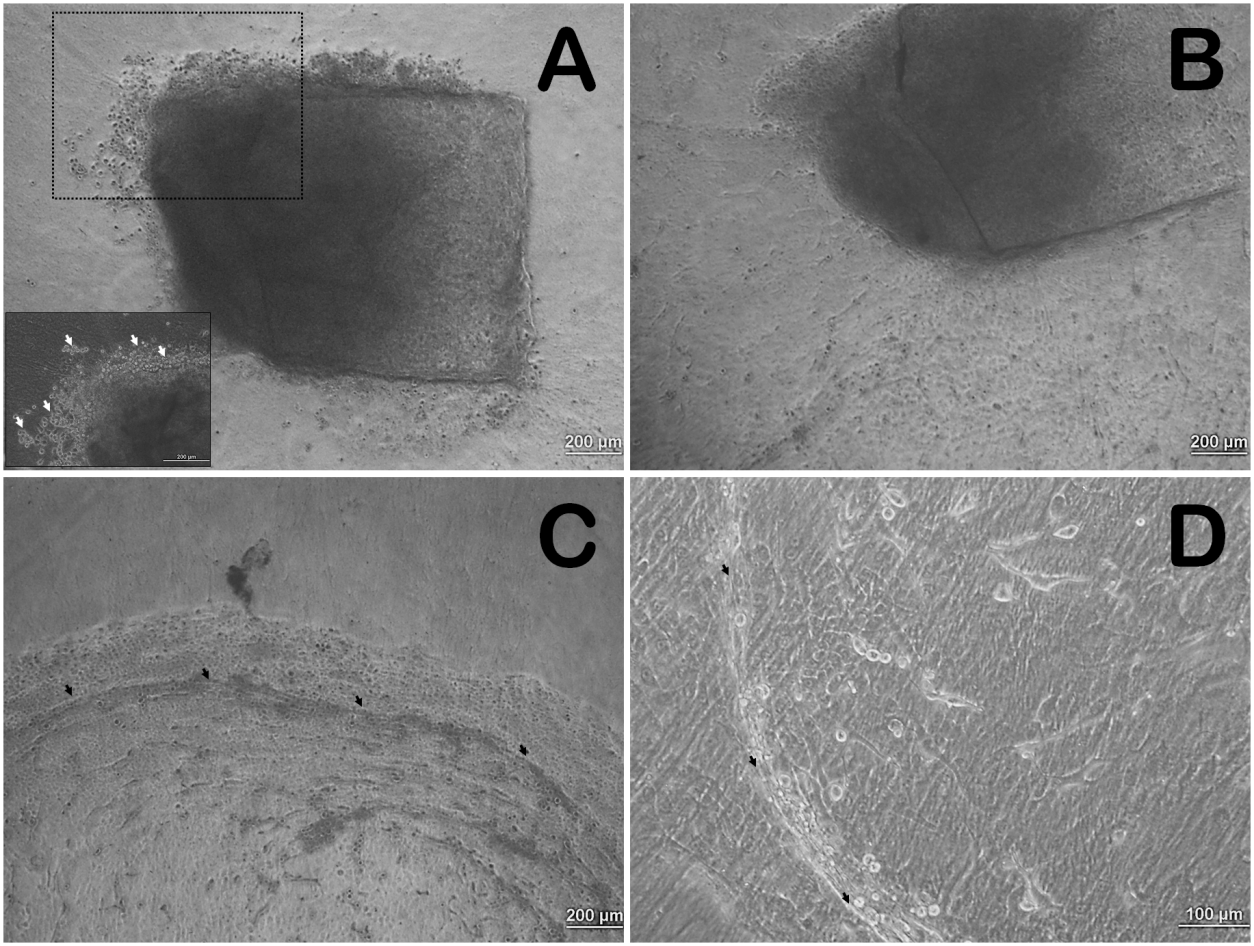
Limbal cell expansion from explant *in vitro*. **A)** Limbal explant culture at day 2 showing round clusters of cells around explant edge. Inset with white arrows showing the same at higher magnification; **B)** Culture at day 3—Disappearance of round cell clusters and expansion of polygonal shaped cells as a sheet. **C & D)** Streak like appearance at the periphery of the outgrowth and the presence of fibroblast shaped stromal cells indicated by black arrows.

**Fig 3:**
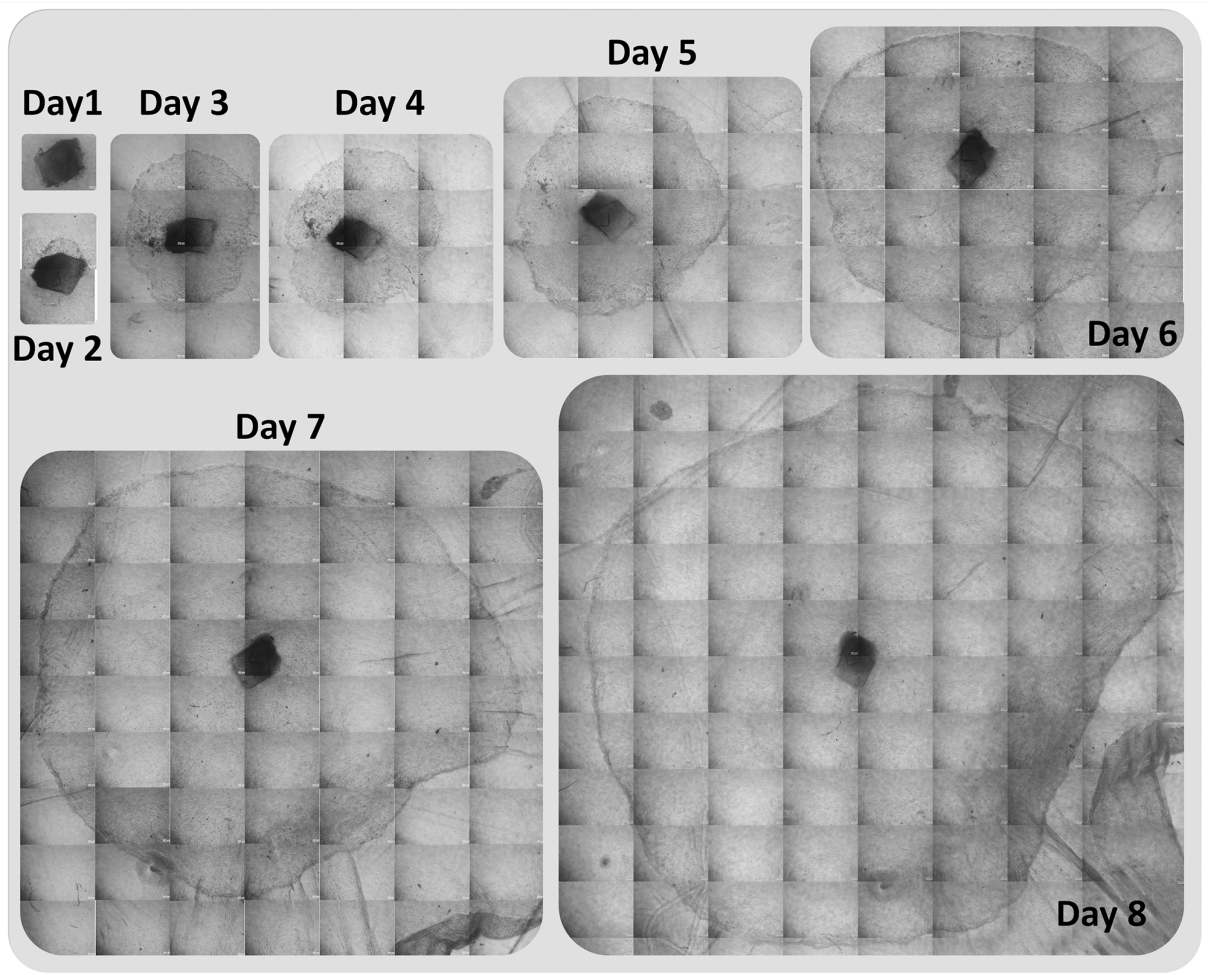
Day wise cell expansion (outgrowth) of a single limbal explant cultured using amniotic membrane as scaffold. Increase in the area of the expansion can be observed with respect to days.

**Fig 4:**
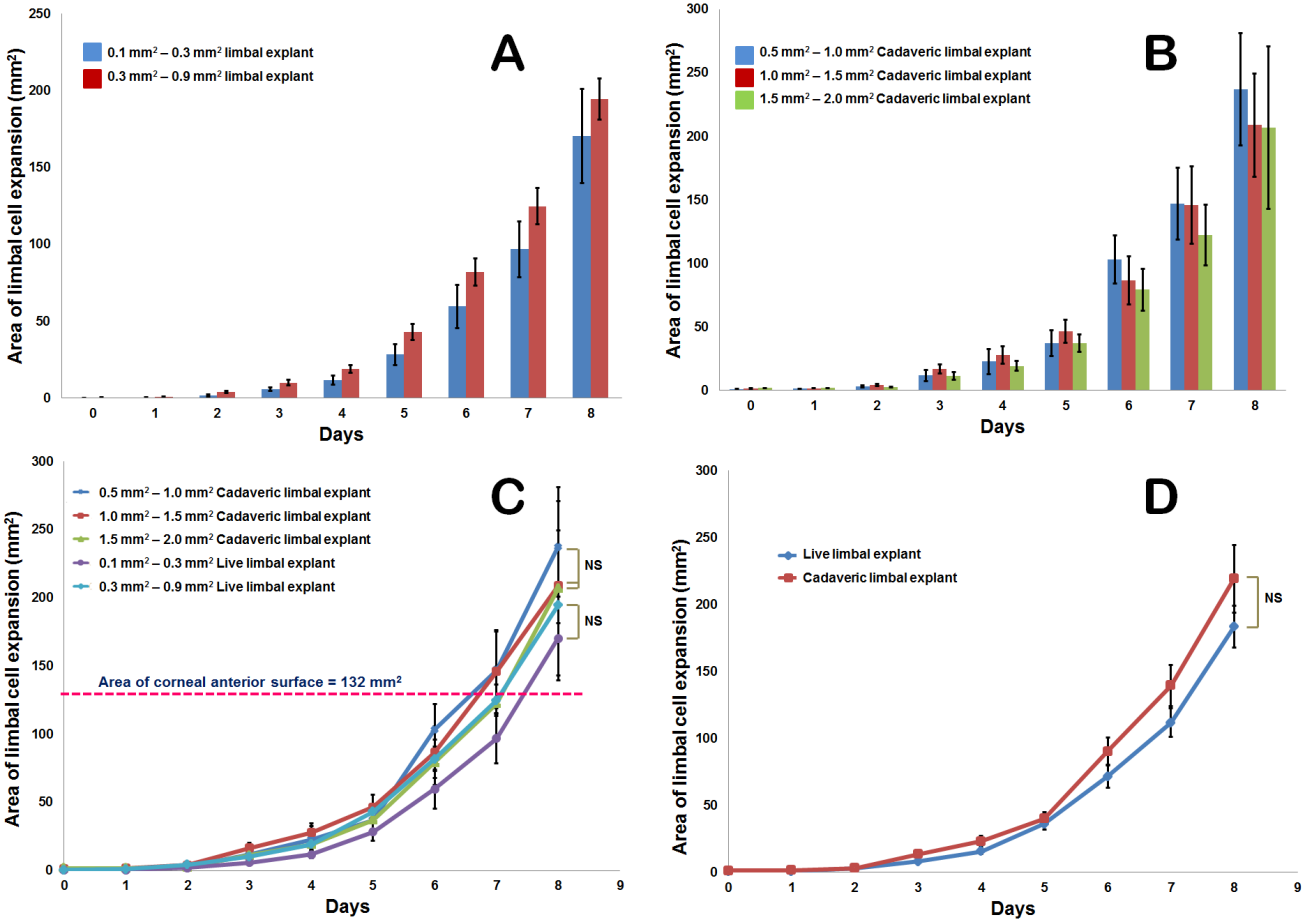
Growth potential of limbal explants. Mean area of live limbal tissue cell expansion with time for live **(A)** and cadaveric **(B)** tissues. Exponential cell expansion can be seen in explants of different size ranges. **C)** Mean area of cell expansion obtained from a single explant of different sizes in live and cadaveric cases. Data shows that a single explant placed on the amniotic membrane has the ability to expand its cells to an area equal to that of anterior corneal surface area (i.e., 132 mm^2^) in a period of 6.5 to 7.5 days. **D)** Expansion potential of cadaveric limbal explants is depicted to be equal to that of live limbal explants. Statistical test used is ‘Linear mixed-effects model fit by maximum likelihood’ with variability of data represented by standard error (SE).

Cultures were maintained in HCE medium until they had reached confluency (Day 8) on the amniotic membrane of 2.5 cm x 2.5 cm. Successful explant expansion from the live tissues obtained was noted to be 80% (n = 8 of 10) (S1B Fig). Cells were observed to grow as an adherent sheet with regular, polygonal shape consisting of a prominent nucleus. The mean average area of expansion for different explant sizes is shown in Table 1. In the cultures, we noted round cell clusters lying on top of the sheet of adherent cells till days 3–4 after which all the cells appeared attached (Fig 2A and 2B). The sheet in most of the cases appeared to expand uniformly around the limbal explant in circular shape and a substantial number of stromal fibroblast-like cells appeared morphologically at the border of the cell sheet expanded in late stage of cultures (Fig 2C and 2D). The whole area of expansion captured with a 4X objective lens was aggregated to measure the growth potential *in vitro* (Fig 3). At confluency i.e. 8 days of explant culture, the area of expansion with respect to time was noted to be exponential (Fig 4A).

**Table 1:** Mean area of expansions with respect to the explant sizes.

Limbal tissue type	Explant size range	Mean expansion area (mm^2^)	p-value
Live	0.1 mm^2^ to 0.3 mm^2^	170.32±81.16	0.183
	0.3 mm^2^ to 0.9 mm^2^	194.46±37.38	
Cadaveric	0.5 mm^2^ to 1.0 mm^2^	237.10±98.52	0.218
	1.0 mm^2^ to 1.5 mm^2^	209.77±90.47	
	1.5 mm^2^ to 2.0 mm^2^	206.91±110.61	

### Potential growth of cadaveric limbus *in vitro*

Cultures maintained in HCE medium until confluency had a successful explant expansion of 60% (n = 6) (S1B Fig). Morphologically, the cells appeared polygonal in shape with regular, well defined borders. They grew as a sheet migrating towards the outer side of the explant without any round cell clusters in the early stages. The expansion was exponential (Fig 4B) with the mean area of expansion for different limbal explant size range shown in Table 1. The results were comparable in the limbal cultures of both live and cadaveric tissues at confluency.

### Minimal explant, growth comparison in live and cadaveric limbal tissue

The minimum amount of limbal tissue required for adequate growth i.e., cell expansion to an area of >132 mm^2^ was found to be in the range of 0.1–0.3 mm^2^ in live and 0.5–1.0 mm^2^ in cadaver *in vitro* (Table 1). Various sizes of the explants obtained from cadaveric and live sources after manually excising with vannas scissors was statistically significant (p≤0.001). Data of the closely related limbal explant size ranges in live (0.3 mm^2^–0.9 mm^2^) and cadaver (0.5 mm^2^–1.0 mm^2^) had respective expansions of 194.46±37.38 mm^2^ and 237.10±98.52 mm^2^ indicating the growth potential of the cadaveric limbal biopsies *in vitro*. Since the sizes of the limbal explants varied among the groups and likewise in between triplicates of the sample, graphs are plotted for the area of expansion against days considering size range as the third variable to identify the growth rate of the cultured tissue (Fig 4C). To indicate the variation among the cell outgrowths from limbal explants, the area of expansion with respect to time for each donor have been plotted (S2A and S2B Fig). At confluency, live limbal explants (size range 0.1 mm^2^–0.9 mm^2^) have shown a mean growth area of 182.39±17.06 mm^2^ in comparison to the cadaveric explants (size range 0.5 mm^2^–2.0 mm^2^) that has a mean growth area of 217.59±16.91 mm^2^ (Fig 4D).

### Proliferating cells in the cultures *in vitro*

**Fig 5:**
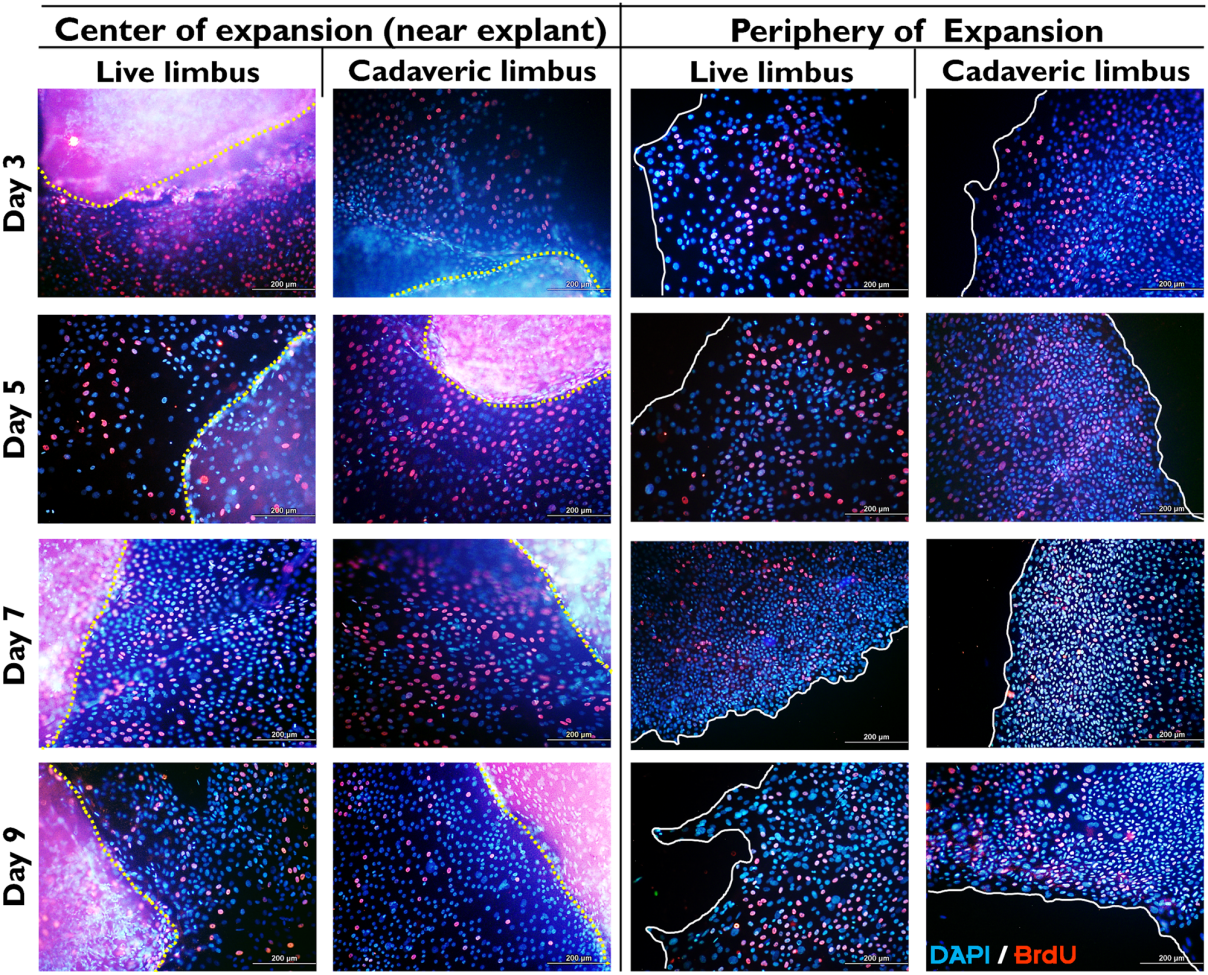
BrdU cell proliferation assay for limbal culture from cadaveric tissue. Cells of the explant expansion showing BrdU (Red) staining denoting cell proliferation compared to the total number of cells represented by nuclear counterstain DAPI (Blue). Yellow dotted lines indicate edge of the explant and white solid lines indicate the periphery of the cell expansion. Explants in left hand pane appear as red patches due to staining of BrdU indicating cell proliferation inside the tissue.

**Fig 6:**
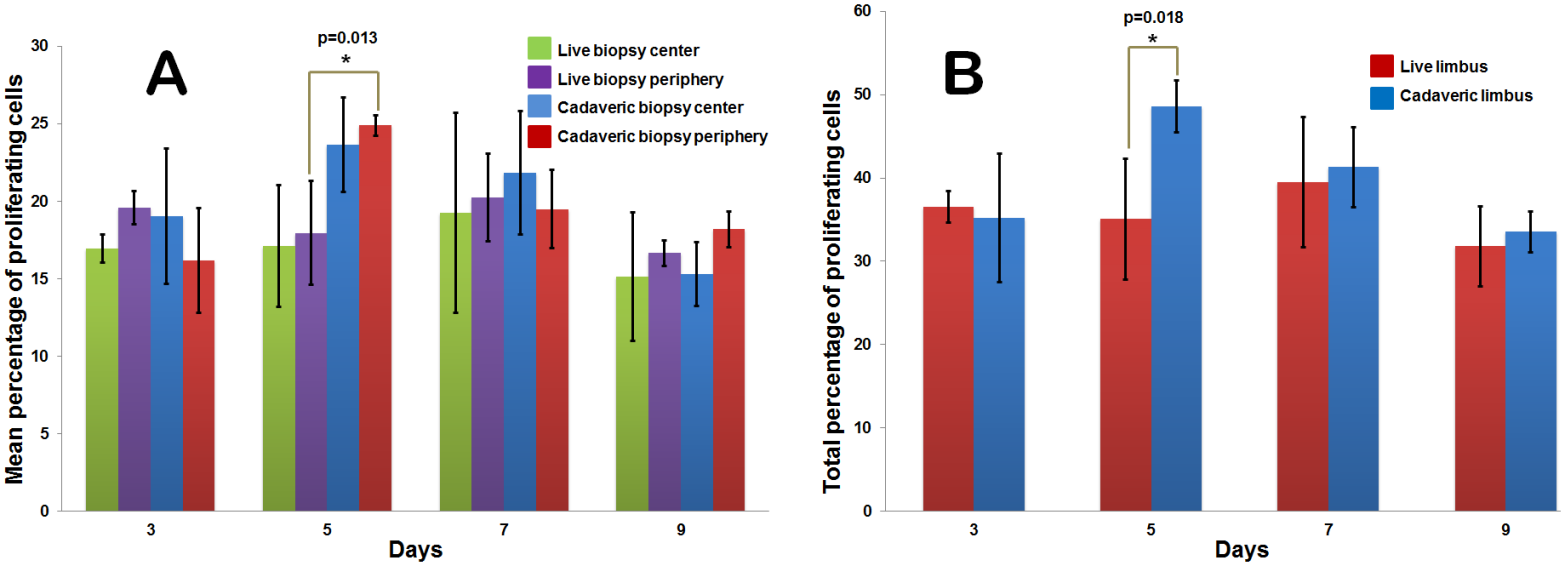
Rate of cell proliferation. **A)** Comparative analysis of mean cell proliferation for live and cadaveric cultures both at center and peripheral positions at different time points. **B)** Total mean percentage of proliferative cells in live and cadaveric cultures. Cadaveric cell proliferation is significantly greater on day 5 at the culture periphery (p = 0.013) in addition to the total proliferation rate (p = 0.018). Statistical test used is ‘Two sample *t*-test’ with variability of data represented by standard error (SE).

Cell proliferation identified by labeling with BrdU appeared as bright red fluorescence dots in the nuclei when stained with anti-BrdU antibody and observed under a fluorescence microscope (Fig 5). The mean percentage of proliferating cells at early and late stages in live and cadaveric limbal explant cultures is shown in Table 2 and Fig 6A. The mean total percentage of proliferating cells at confluency was 31.80±3.81 and 33.49±4.25 for live and cadaveric cultures respectively which was comparable (p = 0.325) (Fig 6B). Besides, the cells in the explants show fluorescence at all the time points indicating their proliferation.

**Table 2:** Percentage of proliferating cells in limbal cultures.

Location	Stage (Day)	Limbal tissue type	% of proliferating cells (30 min BrdU pulse)	p-value
Center of expansion (near the explant)	Early (Day 3)	Live	16.95±1.58	0.465
		Cadaveric	19.02±7.55	
	Late (Day 9)	Live	15.15±7.16	0.462
		Cadaveric	15.31±3.54	
Periphery of expansion	Early (Day 3)	Live	19.56±1.85	0.291
		Cadaveric	16.19±5.85	
	Late (Day 9)	Live	16.65±1.39	0.439
		Cadaveric	18.18±3.01	

### Limbal progenitor and epithelial cell survival

**Fig 7:**
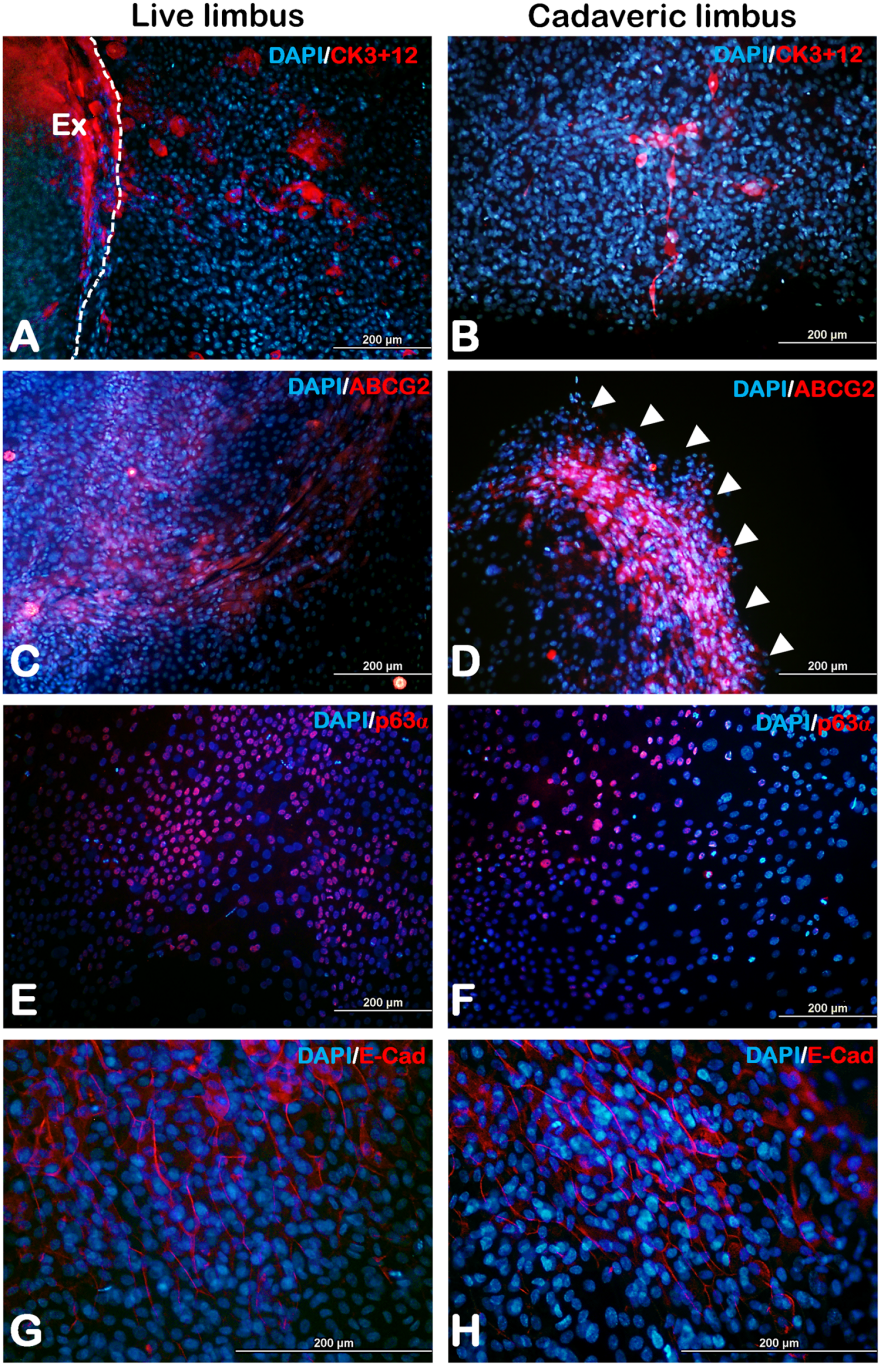
Immunofluorescence. Respective live and cadaveric cultures expressing corneal epithelial cell marker cytokeratin 3+12 **(A&B)**, limbal stem cell markers ABCG2 **(C&D)**, p63α **(E&F)** and epithelial tight junction marker E-Cadherin **(G&H)**. White arrow heads represents the edge of expansion. Ex- Explant; White dotted lines in panel (A) represents the explant border.

At confluency, the limbal epithelial culture grown on amniotic membrane was analyzed for the presence of corneal epithelium or putative limbal stem cells using immunofluorescence. To determine the presence of corneal epithelial cells, expression of cytokeratin 3+12 (Fig 7A and 7B) was assessed which was found to be scattered over the area of cell expansion. In addition to that, to ascertain the presence of limbal progenitors in the culture which would aid in further survival of the culture after transplantation, we had identified the cells expressing ABCG2 which were found specifically nearer to the edge of cell expansion area (Fig 7C and 7D) and p63α expressing cells dispersed across the culture (Fig 7E and 7F). The cells further expressed E-Cadherin denoting the thorough formation of adherens junctions of the epithelial cells (Fig 7G and 7H).

### RT-PCR analysis

Gene expression analysis of the limbal cultures in both cadaveric and live groups had shown similar expression profiles. Limbal cells from both the groups had expressed stem cell markers ABCG2, cytokine IL-6 with respect to the β-Actin expression (S3 Fig).

## Discussion

Our study provided an additional strategy for enhancing success of limbal transplantation by optimizing the explant parameters. We demonstrated for the first time that limbal explant cultures in both cadaveric and live biopsy cases from a single explant can expand its cell growth to an extent beyond the area of the anterior corneal surface i.e., to 132 mm^2^ in a span of 8 days. We show that a minimal amount of 0.3 mm^2^ live or ≥ 0.5mm^2^ cadaver limbal explant is capable of growing and spreading the cells as a sheet on the amniotic membrane culture. Nevertheless, explant size of < 0.5 mm^2^ from cadaver tissue had completely no growth in the cultures compared to the live explant that had adequate cell expansion (S4 Fig). This evidence adds an insight for the minimal donor limbal tissue acquisition during transplantation which is reported to be 4 mm^2^ [13] or at times one clock hour [9] of the limbus from our group. Besides, our data on minimal explant size requirement could be invaluable information particularly in cases of bilateral LSCD or repeated SLET/CLET cases where healthy autologous limbus would be of vital importance. In our study, both live and cadaveric tissue demonstrated similar cell growth potential *in vitro* depending on the successful growth initiation in cadaveric cultures. At culture confluency (day 8), the rate of cell expansion for live and cadaveric tissue was 22.89±1.95 mm^2^/day and 14.61±2.94 mm^2^/day respectively (S5 Fig). In addition to that, the proportion of cell growth from cadaveric limbal cultures was found to be less (60%) in comparison to live cultures (80%) (S1B Fig) This could be attributed to the characteristics such as death to preservation or culture time, age or storage conditions of the cadaver tissue. But, failure to expand *in vitro* for live limbal biopsies in our case (n = 2) is susceptible to the time lapse of biopsy collection.

Conditions of epithelial-mesenchymal transitions have been reported in limbal explant cultures *in vitro* [24, 25] where the limbal epithelial cells of the explant tend to invade in to the stroma before expanding on the amniotic membrane. This could possibly explain the presence of round cell clusters which we have observed in early stages of culture near the explant. However, the sudden disappearance of round cell clusters after day 2 or 3 in our cultures might be due to the mesenchymal epithelial transitions with up regulation of E-Cadherin as described in different studies [26, 27]. Additionally, we have also noticed that the shape of the cell outgrowth from a single explant at all times does not seem to be uniform in all directions beginning from the early stages of the culture. It was noted to be multiform in shape for 68.42% (13/19 explants cultured) of live (S6A Fig) and 50% (8/16 explants cultured) of cadaveric limbus (S6B Fig). In these cultures, the cells in the edges of out-growth are found to be morphologically fibroblastic than that of the epithelial type. This condition tends to be enigmatic since the explants derived from the same tissue tend to behave differently where few explants have uniform cell out growth in all directions and few have multiform growth. Limbal cell proliferation and migration is an important factor as the expansion of the explant culture increases. We have noted that, an overnight (16 hours) pulse labeling of BrdU would render most of the cells to be positive and hence a very minimal time of 30 minutes is chosen to assess the proliferating cells. We have found no significant difference in the percentage of total proliferative cells in live (31.80±3.81) and cadaveric (33.49±4.25) cultures as well as the cells near the explant versus culture periphery. We know that the cells away from the explants are mainly transient amplifying (TA) cells and differentiated cells as reported by Kolli et al [28]. Here, we wish to point out that the early activated stem cells (early TA cells, possibly the progenies of the first few cell division cycles) migrate out of the explants and remain quiescent. They migrate at the leading edge of the epithelial outgrowths, while the progenies lagging behind undergo extensive proliferation. This was shown using various cell cycle and stem cell markers and long-term BrdU label retention assays in one of our recent publications [22]. We believe that these early activated and quiescent TA cells contribute to the stem cell content of the limbal graft. In our study we have found the similar level of expression BrDU positive cells both at far end and near explant, when the limbal explant culture grows successfully (Figs 5 and 6). Transplantation success has shown to contribute to the presence of progenitors specifically the p63 bright [29] and ABCG2 [30] whose stemness is known to be maintained by the human amniotic membrane [31]. Accordingly, we have observed the expression of progenitors (ABCG2, p63α) in our cultures along with the expression of E-Cadherin denoting the formation of tight junctions in epithelium with cells expressing cytokeratin signifying mature corneal or limbal epithelial cells. Gene expression analysis of ABCG2 shows comparable expression pattern of live and cadaveric cultures. Similarly, we have found the IL-6 expression to be similar among live and cadaveric cultures which is reported to be assisting in the epithelial and stromal cell interactions and maintenance of progenitor trait of the cells [32].

Our proposed strategy is especially significant in the context of both SLET and CLET surgery. From our previous experience of SLET [9] which had successful vision restoration, we have explant sizes ranging from 0.04 mm^2^ to 0.5 mm^2^ (p≤0.001) that were manually excised with vannas scissors and placed over the recipient’s affected eye (S7 Fig). This is similar to that of our current *in vitro* study that aimed to investigate the optimal size and expansion ability of a single limbal explant. Additionally, an *in vivo* study by Mittal et al post SLET surgery had described the delayed epithelialization rate with age, size and number of the explants and the reasons were yet unknown [33]. The amount of the limbal tissue required for treating LSCD had always been ambiguous at clinical setting [9, 12] and there seems to be no control over the minimal size of the limbal explant that is excised manually during transplantation. Therefore, a standard value pertaining to the amount of limbus to be acquired for transplantation can be identified based on our study since donor safety after tissue excision is of utmost importance.

Conceptually, we know that variables as such as donor age, the time of death to tissue preservation and the heterogeneous cell populations in the explant are all likely to affect the outcomes of allogeneic SLET. However, the size of the explant could be one of the factors affecting the *in vitro* limbal expansion and subsequent transplantation outcomes. The findings of our study shows that cadaveric explants <0.5 mm^2^ had poor growth potential. However, larger explants (≥ 0.5 mm^2^) had growth rate and proliferative potential similar to the live tissue. These findings could prove to be critical for clinical success especially while attempting cadaveric limbal transplantation. Cadaveric tissues obtained from eye banks or possibly after penetrating keratoplasties could serve as a good source for limbal cells considering the requirement of a larger explant size and younger donor age. In addition to that, the long term effects of the donor site are not well studied and are contradictory where few reports [34, 35] have shown complications in donor sites leading to filamentary keratitis and sub-conjunctival hemorrhage. Clinicians believes that more explant placed on the amniotic membrane during the transplantation will provide a higher number of limbal stem cells and will also compensate for the loss of viability as well as detachment of few explants. Consequently, the need for a bigger explant for better outcome is still debatable. This has to be further validated in animal models on which we are currently working on using the rabbit models of LSCD.

## Supporting information

S1 Fig Successful growth of the limbal culture and age of the donors. **A)** The above graph shows the mean age of the limbal donors was 54.9±10.79 years and 45.3±24.55 years for live and cadaveric respectively. **B)** The above graph shows the percentage of successful growth of the limbal explants *in vitro*. Growth was observed in 80% of live tissue and 60% of the cadaveric tissue.
(TIF)

S2 Fig Donor-wise area of expansion for limbal explant cultured *in vitro*. Mean expansion area for limbal explants obtained from live donors **(A)** and cadaveric donors **(B)**.
(TIF)

S3 Fig Semi-quantitative gene expression. ABCG2 and IL-6 expression in cadaveric and live limbal cultures by reverse transcription PCR. M—100 base pair ladder, C—Cadaveric tissue culture, L—Live tissue culture.
(TIF)

S4 Fig Cell outgrowth for limbal explants <0.5 mm^2^. **A)** No cell expansion was observed in cadaveric explant with less size (0.15 mm^2^) whereas the **B)** live explant of similar size (0.16 mm^2^) had adequate cell expansion at day 8. Dotted white and red lines indicate the area of cell outgrowth and the limbal explant respectively. Arrows shows the amniotic membrane folds. Inset image shows the magnified visual of live limbal explant. Note that, both the pictures are taken at same magnification (40X). However, figure B is a collage to show the growth of the live explant taken at same magnification.
(TIF)

S5 Fig Rate of limbal cell expansion (mm^2^/day). At confluency (8 days), the mean growth rate of live and cadaveric limbal tissues *in vitro* was 22.8 and 14.6 mm^2^/day respectively. ‘T-test’ was used with variability of data represented by standard error (SE).
(TIF)

S6 Fig Limbal explants outgrowth in multiform shape. Outgrowth of cells in multiform shapes from single explant in the cases of live **(A)** and cadaveric **(B)** limbus. Dotted lines of black and white indicate the area of cell outgrowth and the limbal explant respectively.
(TIF)

S7 Fig Limbal explants used in Simple limbal epithelial transplantation. **A)** Limbal explants after 6 days in a patient who underwent autologus SLET surgery. Size of the explants had ranged from 0.04–0.56 mm^2^ **B)** Anterior Segment—Optical Coherence Tomography (AS-OCT) image showing the cross section of the ocular surface of the same patient showing the transplanted limbal explant.
(TIF)

S1 Table List of antibodies. Details of the primary and secondary antibodies used in our study.
(DOCX)
