# Supplementary figures and images for: Optimizing the role of limbal explant size and source in determining the outcomes of limbal transplantation: An in vitro study
Source: PLoS One. 2017 Sep 28;12(9):e0185623. doi: 10.1371/journal.pone.0185623 (PMC5619808; doi:10.1371/journal.pone.0185623)

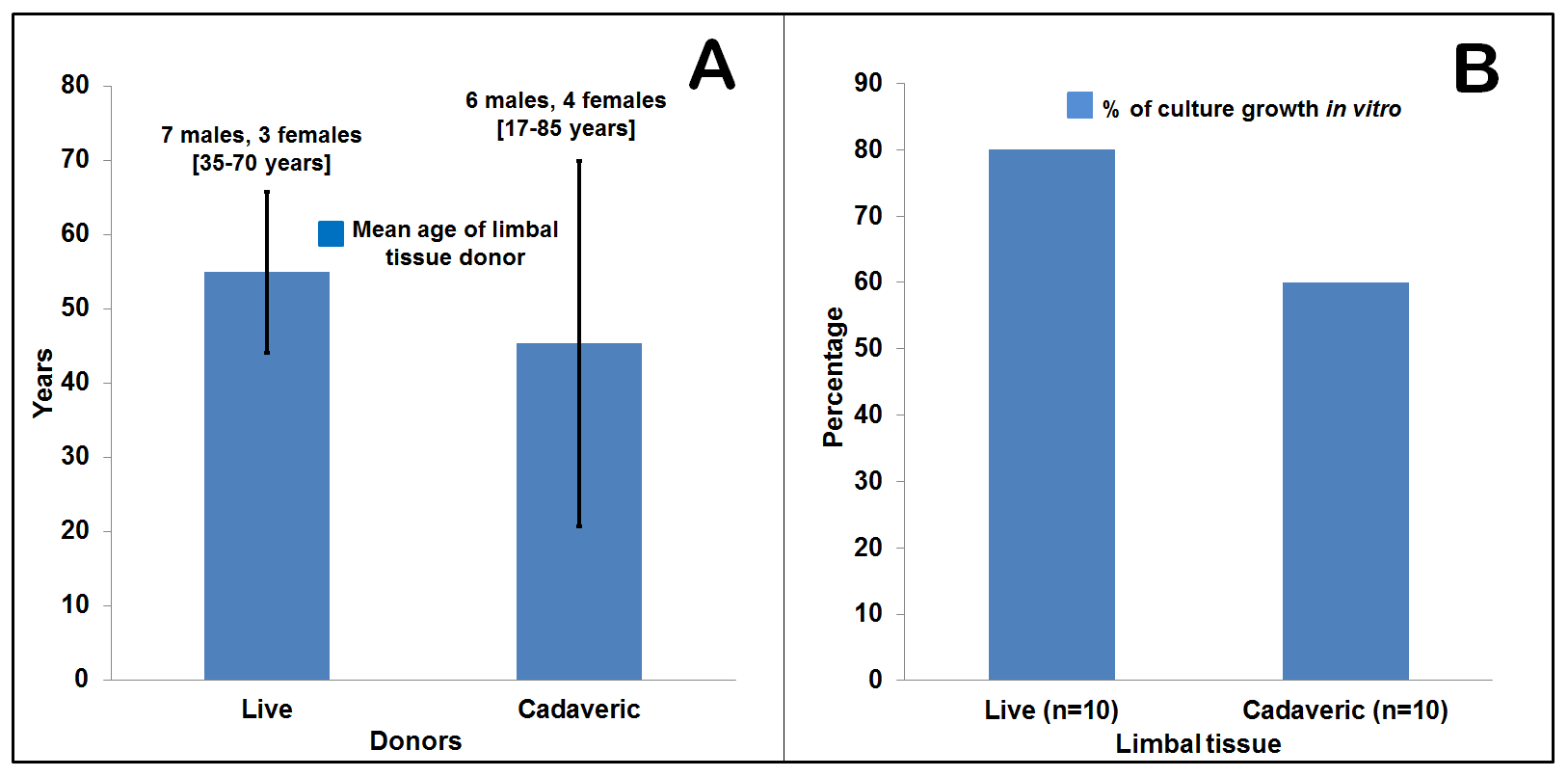

Supplement: S1 Fig — A) The above graph shows the mean age of the limbal donors was 54.9±10.79 years and 45.3±24.55 years for live and cadaveric respectively. B) The above graph shows the percentage of successful growth of the limbal explants in vitro. Growth was observed in 80% of live tissue and 60% of the cadaveric tissue. (TIF) [file pone.0185623.s001.tif]

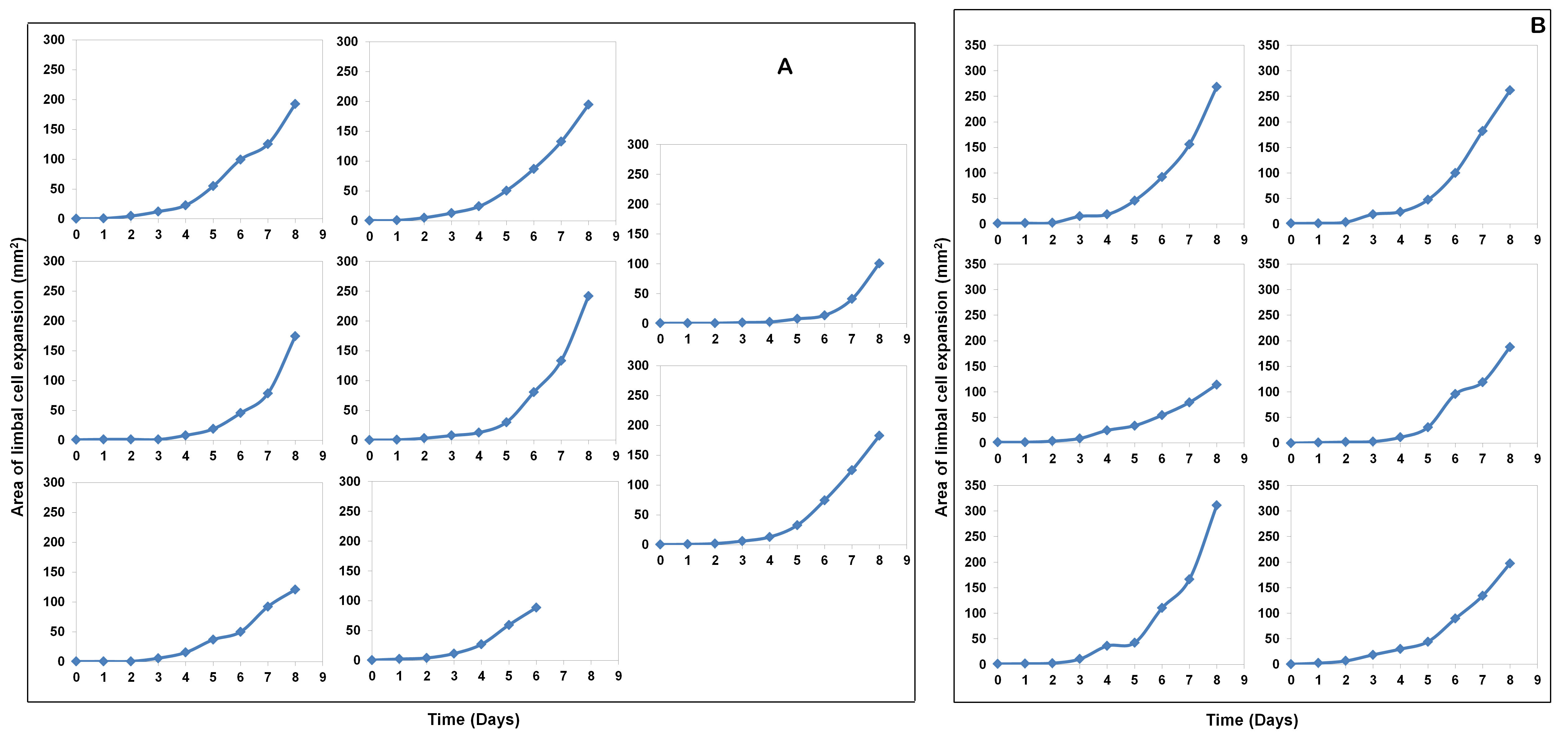

Supplement: S2 Fig — Mean expansion area for limbal explants obtained from live donors (A) and cadaveric donors (B). (TIF) [file pone.0185623.s002.tif]

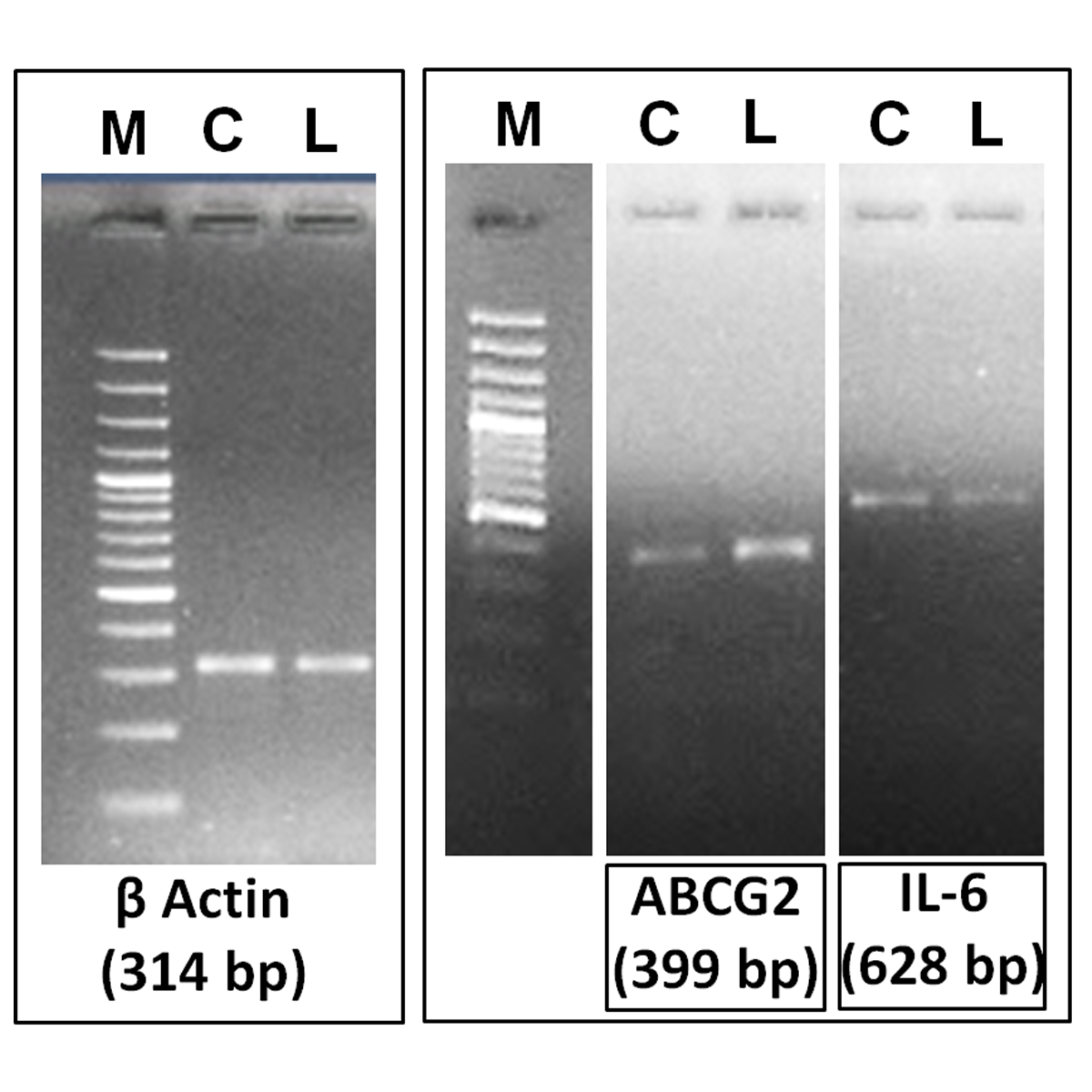

Supplement: S3 Fig — ABCG2 and IL-6 expression in cadaveric and live limbal cultures by reverse transcription PCR. M—100 base pair ladder, C—Cadaveric tissue culture, L—Live tissue culture. (TIF) [file pone.0185623.s003.tif]

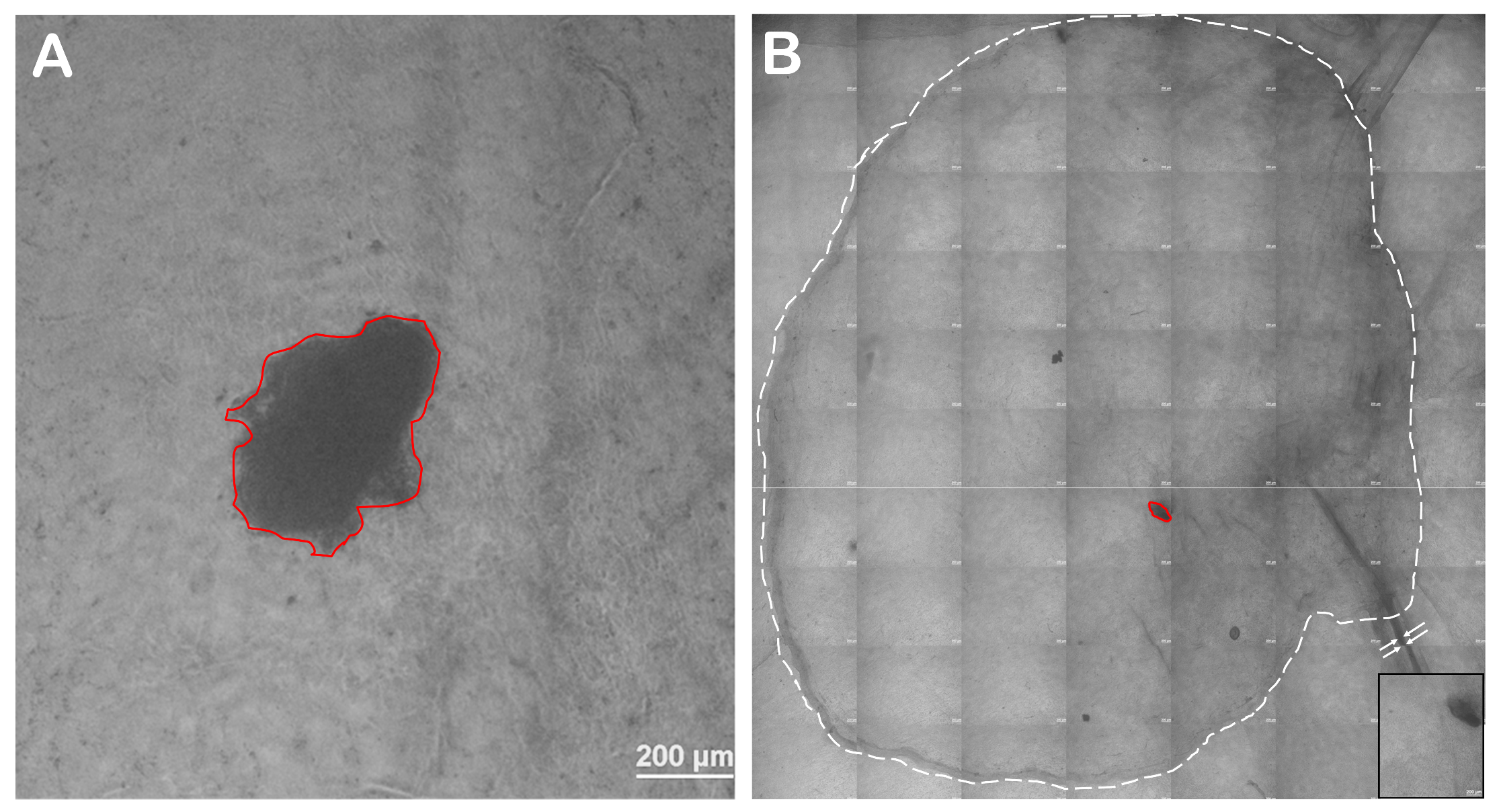

Supplement: S4 Fig — A) No cell expansion was observed in cadaveric explant with less size (0.15 mm2) whereas the B) live explant of similar size (0.16 mm2) had adequate cell expansion at day 8. Dotted white and red lines indicate the area of cell outgrowth and the limbal explant respectively. Arrows shows the amniotic membrane folds. Inset image shows the magnified visual of live limbal explant. Note that, both the pictures are taken at same magnification (40X). However, figure B is a collage to show the growth of the live explant taken at same magnification. (TIF) [file pone.0185623.s004.tif]

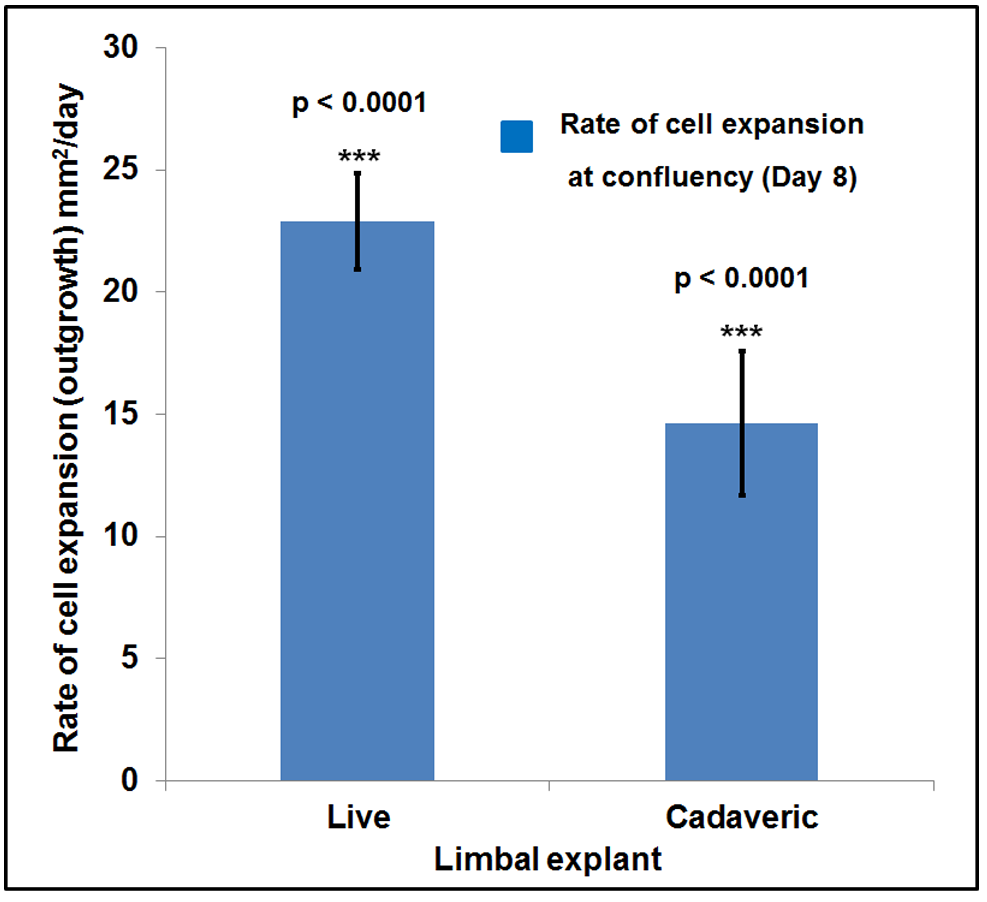

Supplement: S5 Fig — At confluency (8 days), the mean growth rate of live and cadaveric limbal tissues in vitro was 22.8 and 14.6 mm2/day respectively. ‘T-test’ was used with variability of data represented by standard error (SE). (TIF) [file pone.0185623.s005.tif]

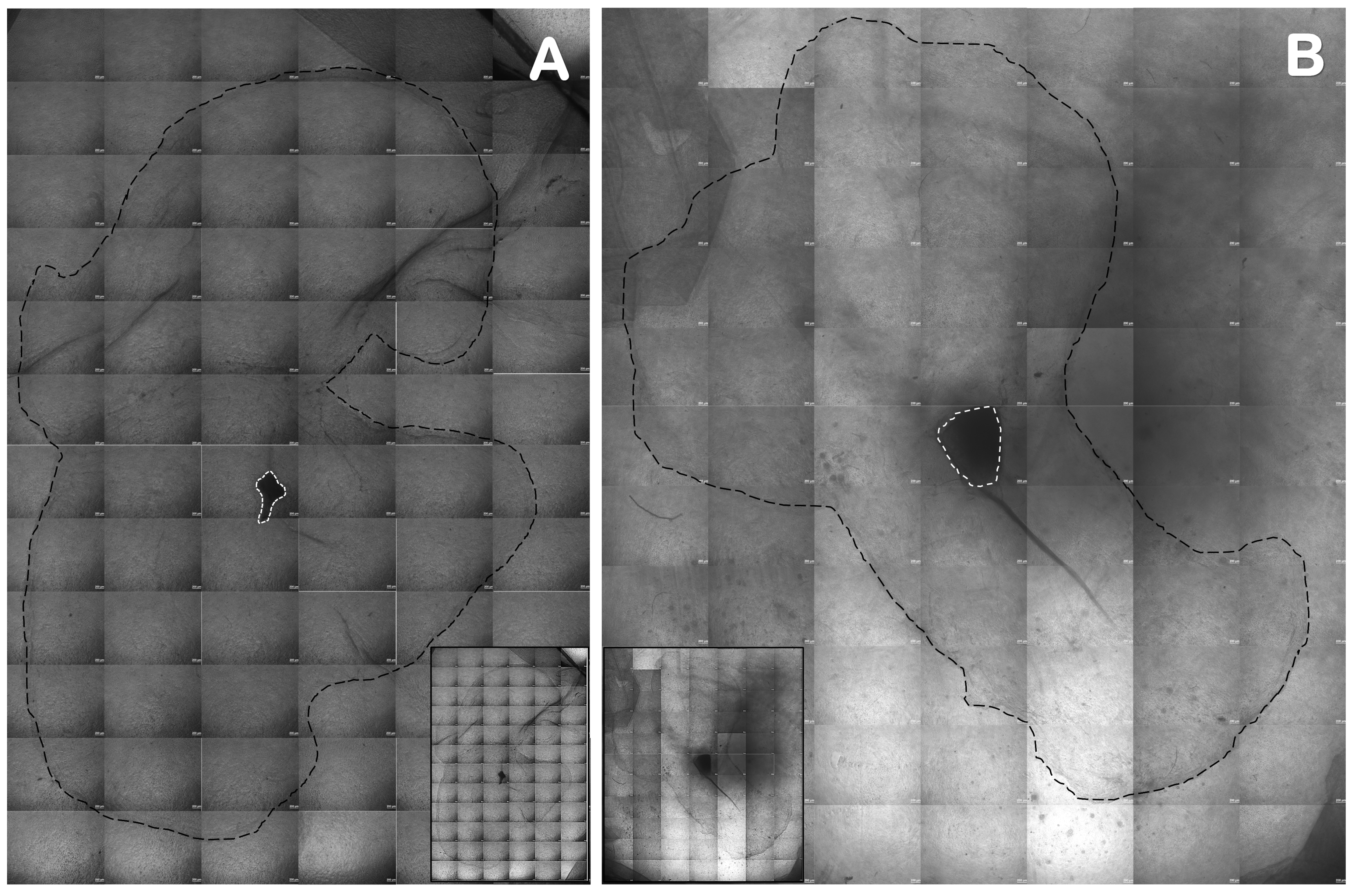

Supplement: S6 Fig — Outgrowth of cells in multiform shapes from single explant in the cases of live (A) and cadaveric (B) limbus. Dotted lines of black and white indicate the area of cell outgrowth and the limbal explant respectively. (TIF) [file pone.0185623.s006.tif]

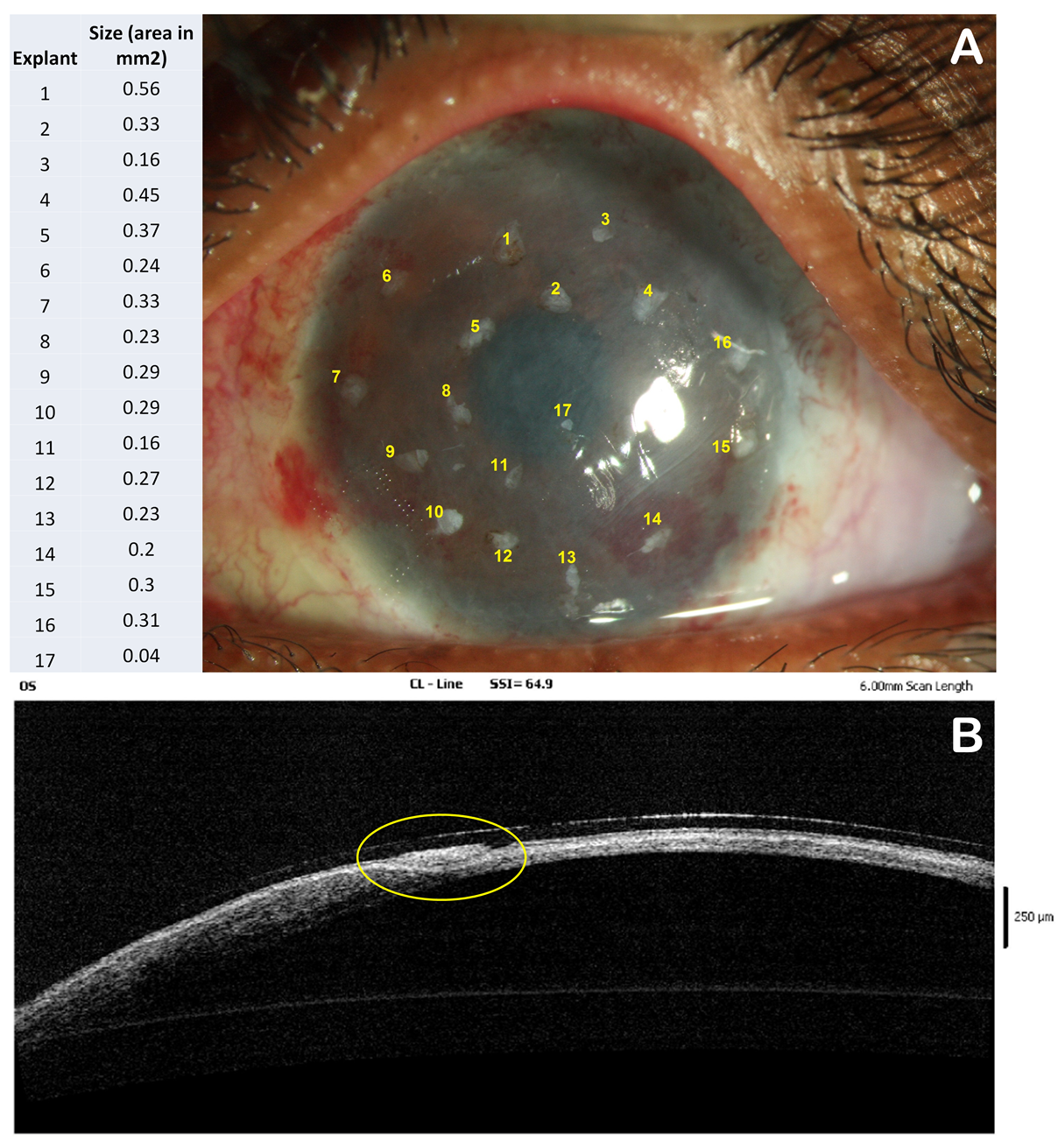

Supplement: S7 Fig — A) Limbal explants after 6 days in a patient who underwent autologus SLET surgery. Size of the explants had ranged from 0.04–0.56 mm2 B) Anterior Segment—Optical Coherence Tomography (AS-OCT) image showing the cross section of the ocular surface of the same patient showing the transplanted limbal explant. (TIF) [file pone.0185623.s007.tif]
