# Supplementary material for: Optimizing the role of limbal explant size and source in determining the outcomes of limbal transplantation: An in vitro study
Source: PLoS One. 2017 Sep 28;12(9):e0185623. doi: 10.1371/journal.pone.0185623 (PMC5619808; doi:10.1371/journal.pone.0185623)
Supplement: S1 Table — Details of the primary and secondary antibodies used in our study. (DOCX) [file pone.0185623.s008.docx]

**S1 Table: List of antibodies**

| **Antibody** | **Make** | **Catalog** | **Dilution** |
| --- | --- | --- | --- |
| **Primary antibodies (Unconjugated)** | | | |
| CK 3+12 | Abcam, Cambridge, UK | ab68260 | 1:100 |
| ABCG2 | Santa Cruz Biotechnology, Texas, USA | SC-18841 | 1:10 |
| p63α | Cell Signaling Technology, MA, USA | #4892 | 1:50 |
| E-Cadherin | Abcam, Cambridge, UK | ab1416 | 1:100 |
| BrdU | Santa Cruz Biotechnology, Texas, USA | SC-32323 | 1:100 |
| **Secondary antibodies (Fluorescence conjugated)** | | | |
| Alexa Fluor 594 Goat anti-mouse IgG H+L | Invitrogen, CA, USA | A-11005 | 1:500 |
| Alexa Fluor 594 Goat anti-rabbit IgG H+L | Invitrogen, CA, USA | A-11012 | 1:500 |

Details of the primary and secondary antibodies used in our study.
